# Screening practices and risk assessment for maculopathy in pentosan polysulfate users across different exposure levels

**DOI:** 10.1038/s41598-024-62041-y

**Published:** 2024-05-17

**Authors:** Hyeon Yoon Kwon, Jiyeong Kim, Seong Joon Ahn

**Affiliations:** 1 Department of Ophthalmology, Hanyang University Hospital, Hanyang University College of Medicine, 222-1 Wangsipli-Ro, Seongdong-Gu, Seoul, 04763 Republic of Korea; 2 Department of Pre-Medicine, College of Medicine, and Biostatistics Lab, Medical Research Collaborating Center (MRCC), Hanyang University, Seoul, Republic of Korea

**Keywords:** Diseases, Eye diseases

## Abstract

In this population-based cohort study, we investigated screening practices for maculopathy and incidences of specific macular/retinal conditions in pentosan polysulfate (PPS) users and assessed the relationship between these outcomes and drug exposure levels. Using a health claims database that covers approximately 50 million Koreans, we identified 138,593 individuals who were prescribed PPS between 2010 and 2021. For the 133,762 PPS users who initiated therapy between 2012 and 2021, the cumulative PPS dose for each participant was evaluated, and based on their cumulative PPS dose, patients were categorized into the high-risk (≥ 500 g), low-risk (50–500 g), and minimal exposure (< 50 g) groups. We analyzed the performance and methods of these examination methods used between 2018 and 2021 and compared them among cumulative dose groups to determine whether high-risk users underwent maculopathy screening more frequently or appropriately. We assessed the cumulative incidence of overall macular degeneration and maculopathy excluding common macular diseases following PPS therapy initiation. Most PPS users (99.7%) received a cumulative PPS dose < 500 g and the high- and low-risk groups comprised 445 (0.3%) and 22,185 (16.6%) patients, respectively. During the study period, monitoring examinations were conducted in 52.6% and 49.4% of high- and low-risk patients, respectively, revealing no significant difference between the two groups (P = 0.156). No significant differences were observed in the annual percentages of patients receiving ophthalmic examinations between the high- and low-risk groups (all P > 0.05). The cumulative incidences of overall macular degeneration and maculopathy excluding common macular diseases in high-risk users were 19.3% and 9.0%, respectively, which were significantly different from those of low-risk users (both P < 0.001). Multivariate Cox regression analysis revealed significantly higher risks of maculopathy excluding common macular diseases in the low- (Hazard ratio [HR] of 1.55 [95% CI 1.13–2.12]) and high-risk groups (HR of 1.66 [95% CI 1.22–2.27]) compared to the minimal exposure group. Our findings suggest a need for increased emphasis on PPS maculopathy screening in high-risk patients, highlighting raising awareness regarding exposure-dependent risks and the establishment of screening guidelines.

## Introduction

Pentosan polysulfate (PPS) is a semi-synthetic polysulfated xylan that has been widely used for the treatment of interstitial cystitis/bladder pain syndrome, a chronic inflammatory condition of the bladder that causes urinary frequency, urgency, and irritation^1,2^. In recent years, there has been a growing concern regarding the retinal toxicity in the PPS users^3–5^. Specifically, maculopathy associated with PPS use, termed PPS maculopathy (PPM), is characterized by retinal pigmentary changes, outer retinal atrophy, and permanent vision loss in advanced stages^3–8^.

The exact mechanism by which PPS contributes to maculopathy remains unclear^3,8–11^. PPS induces toxicity primarily in the retinal pigment epithelium (RPE), leading to subsequent photoreceptor damage^5,10^. Recent studies revealed a prevalence rate of 12.7% for PPM among patients administered with cumulative doses of PPS between 500 and 999 g^6^, with the prevalence increasing to 50% among those with a cumulative dose > 1500 g^7^. Some single-center studies have demonstrated a significant association between cumulative PPS dosage and maculopathy^6,12^, although not all macular abnormalities have been conclusively attributed to PPS-induced toxicity^12^. Notably, cases of maculopathy have primarily been documented in Caucasian populations, with very limited study in other ethnic groups^6,7,9^. Moreover, the establishment and comprehensive investigation of maculopathy screening practices in PPS users is lacking.

Although PPS was introduced in South Korea in 2003, no instances of PPS-induced maculopathy have been documented. This could be due to the limited number of PPS users in Korea or undiagnosed maculopathy stemming from insufficient screening^13^. Additionally, in Korea, no guidelines or recommendations are available regarding maculopathy screening provided by urologists or ophthalmologists. This lack of standardized screening practices may contribute to the unawareness of the risk of maculopathy and the requirement for their screening in PPS users and lead to wide variability in screening practices among physicians. Accordingly, recommendations regarding monitoring examinations should be established, ideally based on the risk of retinopathy for PPS users, as is done for other toxic retinopathies, such as hydroxychloroquine retinopathy^14,15^.

Herein, we assessed the risk of maculopathy in three groups of PPS users categorized based on their level of drug exposure. By investigating the maculopathy screening practices and comparing them between high- and low-risk users, our goal was to ascertain whether high-risk users underwent more frequent and appropriate maculopathy screenings than low-risk users. From our data, we intended to address how to enhance PPM monitoring for at-risk users.

## Methods

### Subjects

**Figure 1:**
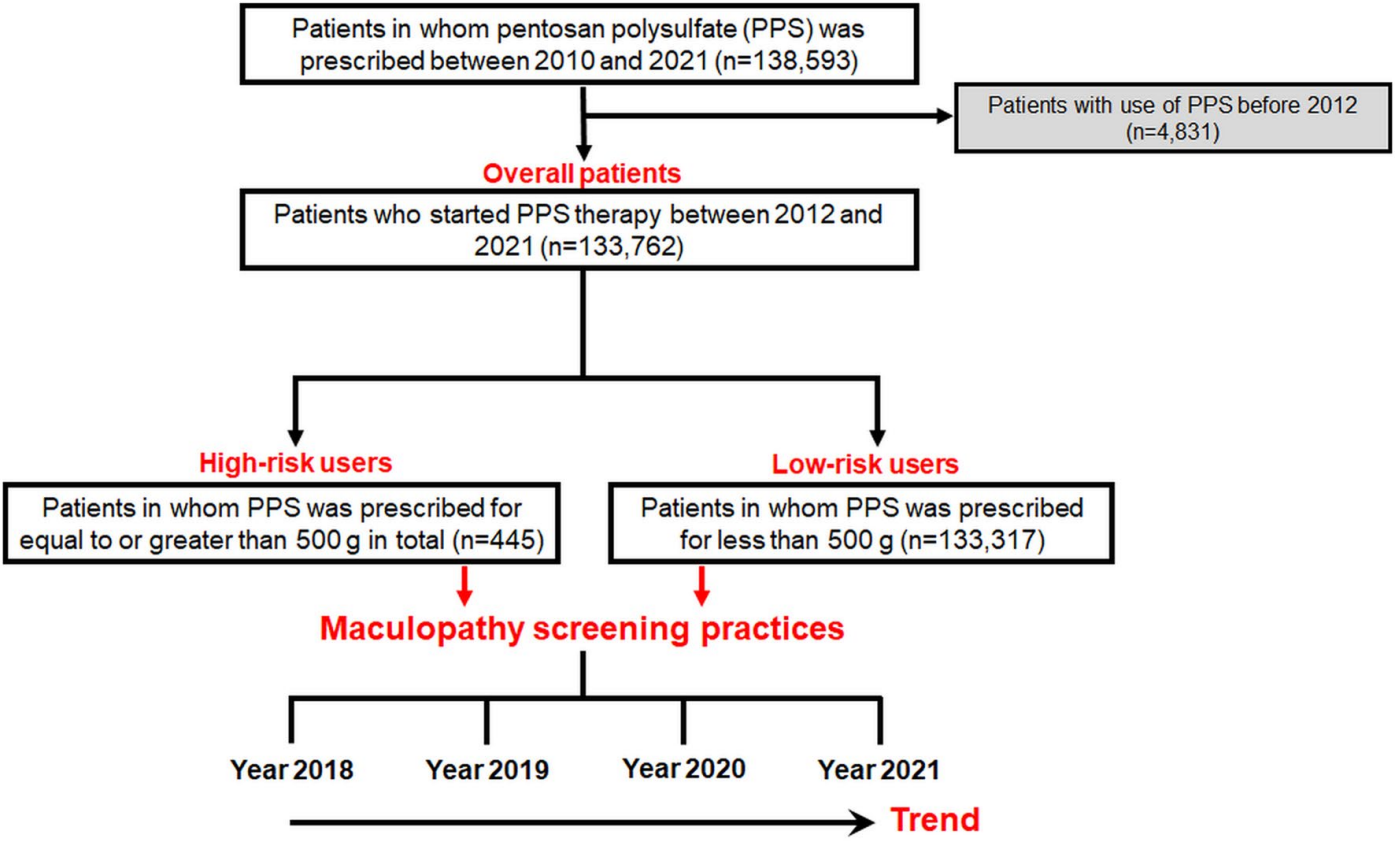
Flowchart illustrating the inclusion and exclusion criteria employed in this study, along with the resulting study population after applying these criteria. Terms used and analyses conducted in this study are highlighted in red text for reference.

This nationwide population-based retrospective cohort study utilized the Health Insurance Review and Assessment (HIRA) database of South Korea, which contains comprehensive information on the medical diagnoses, examinations performed, prescription records, visit dates, and demographic characteristics of approximately 50 million individuals in South Korea. Diagnostic codes in the HIRA database were derived from the Korean Standard Classification of Diseases (KCD), 7th and 8th Revision, with slight adjustments from the International Statistical Classification of Diseases and Related Health Problems, Tenth Revision (ICD-10). Using health claims data from the database, we identified PPS users between January 1, 2010, and December 31, 2021, who represented patients at risk of PPM herein. Among the 138,593 PPS users, patients who had used PPS before January 1, 2012, were excluded from the analysis to ensure that the included patients initiated their therapy after January 1, 2012, for accurate assessment of treatment duration. Following the approach adopted by previous studies on drug-induced retinal toxicity utilizing health claim databases, we assumed that patients without PPS usage for 2 consecutive years (between January 1, 2010, and December 31, 2011) likely did not receive PPS prior to January 2010 due to the chronic nature of PPS use^5,13,16^. Additionally, patients who underwent fundus or macular examinations (fundoscopy, optical coherence tomography [OCT], fundus autofluorescence [FAF], visual field [VF], fluorescein angiography [FA], or electroretinography [ERG]) for any preexisting ophthalmic disease (ICD-10 codes H00-H59) before the initial use of PPS were excluded to eliminate visits scheduled to monitor preexisting ocular conditions. The detailed inclusion and exclusion criteria and the number of patients included are shown in Fig. 1. The study protocol was approved by the Institutional Review Board of Hanyang University Hospital (IRB File no. 2023-01-003) and conducted in accordance with the principles of the Declaration of Helsinki. The need for informed consent was waived by the Institutional Review Board of Hanyang University Hospital due to the retrospective nature of the study and the use of de-identified data.

### Definitions and evaluation

Herein, we employed several definitions based on previous studies. Considering that the risk of maculopathy exceeded 10% in individuals with a cumulative dose of 500–999 g and increased to 50% in those with a cumulative dose exceeding 1500 g^6,8^, high-risk users in our cohort were defined as patients who received a cumulative PPS dose ≥ 500 g, given the absence of patients with cumulative doses > 1500 g in our cohort. This threshold aligns with previous recommendations for regular screening^5,7^. Low-risk users, indicative of individuals with a potential risk of maculopathy based on the minimum dose reported in the literature for either definite or suspected maculopathy but with a comparatively lower risk^5,17,18^, were those with cumulative PPS doses ranging between 50 and 500 g. Finally, the minimal exposure group comprised individuals with a cumulative dose < 50 g, equivalent to PPS usage for less than 6 months at a conventional daily dose of 300 mg. Although the threshold dose requiring PPS maculopathy screening is yet to be established in the literature, one report suggested a threshold of 6 months for the recommendation of baseline examination^5^, implying that PPS use for < 6 months may not induce maculopathy and thus, is not sufficient to recommend screening. Therefore, the minimal exposure group was defined to indicate individuals with an extremely low risk of maculopathy, for whom screening for PPS-induced maculopathy may not be necessary.

In this study, we used diagnostic codes to identify the specific macular conditions that occurred after the initial administration of PPS (Supplemental Table 1). However, the diagnostic code for toxic maculopathy (KCD code: H35.37) was only available within a limited timeframe (up to 2020) in the database. Given that reports of PPS-induced maculopathy began to emerge in November 2018^3^, we broadened our definitions for PPS-associated maculopathy to include a more comprehensive range of macular conditions encompassing overall macular degeneration and maculopathy excluding common macular diseases (Supplemental Table 1).

The monitoring examination referred to the ophthalmic examination for macular or retinal evaluations with respect to structure and function, including fundoscopy, fundus photography, OCT, FAF, VF, FA, and full-field or multifocal ERG, performed for PPS users after the initiation of PPS. Annual monitoring from a 500 g cumulative dose was defined as monitoring examination within 1 year from the timing of the cumulative dose of 500 g. Recent monitoring was defined as monitoring examination during the 1-year time frame before the end date of the observation period. Monitoring with appropriate imaging was defined as that performed with OCT or FAF, as recommended in the literature^3,7,9^. Near-infrared reflectance, a potentially valuable tool for screening PPM^7^, could not be assessed in our study because of the unavailability of its performance code in our health claim database. The percentages of patients receiving annual monitoring from a 500 g cumulative dose and those receiving recent monitoring were evaluated.

### Analysis

Descriptive statistics were used to analyze the demographic and baseline clinical characteristics of the study population, including age, sex, medical indications for PPS use, and the cumulative dose of PPS. Statistics were also used for the performance data and modalities used for the monitoring examinations. Categorical variables were presented as frequencies and percentages, whereas continuous variables were reported as mean ± standard deviation.

Inferential statistics were used to analyze the association between cumulative doses and practice patterns. Chi-square tests were used to compare the proportions of high- and low-risk patients who underwent monitoring examinations. The Cochran–Armitage trend test was employed to assess the statistical significance of the trends in the annual percentage of patients receiving ophthalmic (retinal) examinations in both the low- and high-risk groups across different years. Student’s t-tests were used to compare the number of monitoring examinations performed during the study period.

We calculated the cumulative incidences of overall macular degeneration and maculopathy excluding common macular diseases, using Kaplan–Meier curves. To assess the impact of different risk groups on the development of macular conditions among PPS users, we computed hazard ratios (HRs) using Cox proportional hazards models, which were adjusted for potential confounding factors, including age, sex, and systemic diseases, such as diabetes mellitus (DM) and hypertension (HTN). All P-values were obtained from two-sided tests, and statistical significance was set at P < 0.05. Statistical analyses were conducted using the SAS Enterprise Guide Software, version 7.1 (SAS Institute Inc.).

## Results

### Baseline characteristics and drug exposure of the study population

The demographic and clinical characteristics of the study population are summarized in Table 1. A total of 133,762 individuals constituted the overall user cohort, of which 445 were categorized as high-risk users. In the high-risk group, 58.0% were men and 42.0% were women, whereas in the low-risk group, 50.8% were men and 49.2% were women. The mean ages of high- and low-risk users were 63.9 ± 11.1 and 62.3 ± 13.9 years, respectively. Notably, high- and low-risk users were predominantly aged > 50 years. Moreover, the primary indication for PPS was interstitial cystitis, which accounted for 90.8% and 93.7% of the high- and low-risk users, respectively. PPS was predominantly prescribed by urologists (98.4% and 96.4% of high- and low-risk patients, respectively), as well as internal medicine specialists (0.5% of high-risk patients and 1.4% of low-risk patients), and other specialists (1.1% of high-risk patients and 2.2% of low-risk patients).

**Table 1:** Demographic and clinical information of the pentosan polysulfate (PPS) users categorized by exposure levels: high-risk (> 500 g), low-risk (50–500 g), and minimal exposure (< 50 g).

Characteristics	High-risk (n = 445)	Low-risk (n = 22,185)	Minimal exposure (n = 111,131)
Sex			
Male: female	258 (58.0%):187 (42.0%)	11,273 (50.8%):10,912 (49.2%)	39,187 (35.3%):71,944 (64.7%)
Mean age (± SD), years	63.9 ± 11.1	62.3 ± 13.9	56.9 ± 16.0
< 30	0	596 (2.7%)	7488 (6.7%)
30–39	12 (2.7%)	973 (4.4%)	10,255 (9.2%)
40–49	35 (7.9%)	2214 (10.0%)	16,174 (14.6%)
50–59	101 (22.7%)	4625 (20.9%)	24,811 (22.3%)
60–69	139 (31.2%)	6330 (28.5%)	26,158 (23.5%)
70–79	133 (29.9%)	5553 (25.0%)	18,892 (17.0%)
≥ 80	25 (5.6%)	1894 (8.5%)	7353 (6.6%)
Indication for PPS use			
Interstitial cystitis	404 (90.8%)	20,780 (93.7%)	101,313 (91.2%)
Other cystitis	14 (3.2%)	597 (2.7%)	4888 (4.4%)
Neuromuscular dysfunction of the bladder	14 (3.2%)	490 (2.2%)	2391 (2.2%)
Others	13 (2.9%)	318 (1.4%)	2539 (2.3%)
Medical specialties prescribing PPS			
Urology	438 (98.4%)	21,393 (96.4%)	93,988 (84.6%)
Internal medicine	2 (0.5%)	304 (1.4%)	3483 (3.1%)
Others	5 (1.1%)	488 (2.2%)	13,660 (12.3%)
Cumulative dose of PPS (± SD), g	635.5 ± 128.3	129.4 ± 89.5	11.3 ± 11.9

*SD* standard deviation.

On average, the cumulative dose of PPS received was 635.5 ± 128.3 g for high-risk users, whereas low-risk patients were administered a significantly smaller mean cumulative dose of 129.4 ± 89.5 g. Among 445 high-risk users, 10 (2.2%) had cumulative doses greater than 1000 g. The number of new high-risk users and cumulative high-risk users each year from 2018 to 2021 are presented in Table 2, which reveals a significant increase in the cumulative number of high-risk users over time. This increase was primarily driven by the rapid growth in the number of new high-risk users each year, from 60 in 2018 to 161 in 2021.

**Table 2:** Yearly trend of numbers of overall pentosan polysulfate (PPS) users, new high-risk (> 500 g in cumulative dose) users at the year, and cumulative number of high-risk users from 2018 to 2021.

Year	Number of overall PPS users at the year	Number of new high-risk PPS users at the year	Cumulative number of high-risk users
2018	25,726	60	97
2019	30,438	63	160
2020	32,546	124	284
2021	40,451	161	445

### Maculopathy in high- and low-risk patients

**Figure 2:**
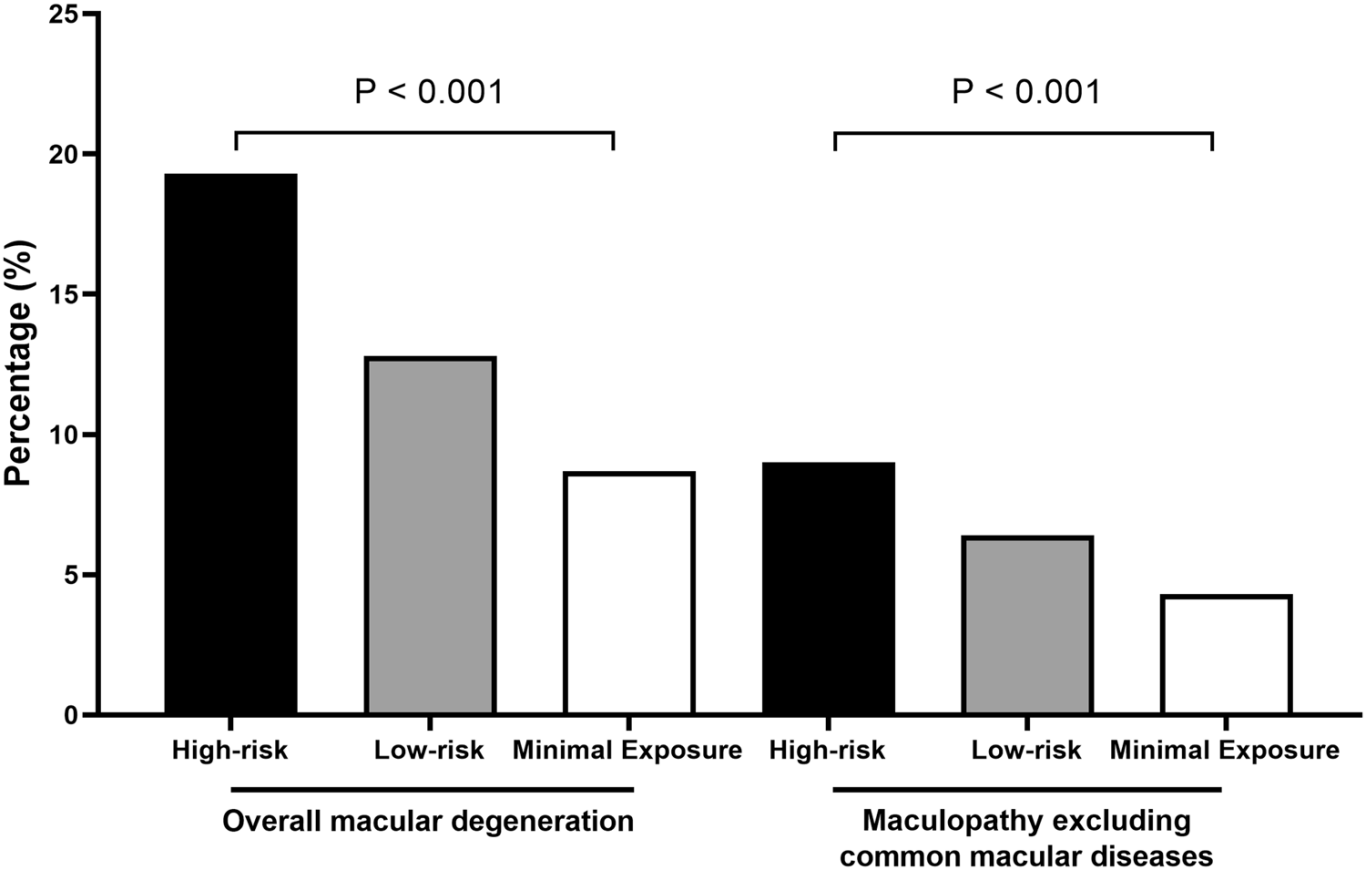
Cumulative incidence of maculopathy in high-and low-risk pentosan polysulfate users.

Figure 2 illustrates the cumulative incidences of overall macular degeneration and maculopathy excluding common macular diseases in the high- and low-risk groups. These results highlighted a substantial disparity in the incidences of maculopathy between the two groups. Among high-risk patients, 19.3% had overall macular degeneration during the study period, in contrast to the lower cumulative incidence of 12.8% in low-risk patients. Moreover, the diagnostic codes for maculopathy, excluding common macular diseases such as age-related macular degeneration, epiretinal membrane, and macular hole, were identified in 9.0% of high-risk patients, compared to 6.4% and 4.3% in the low-risk and minimal exposure groups, respectively. The P-values of < 0.001 for both comparisons underscored the significance of risk groups separated by the cumulative dose of PPS in the cumulative incidence of overall macular degeneration and maculopathy excluding common macular diseases.

### Screening practices in high- and low-risk patients

Table 3 provides a comprehensive overview of the screening practices used to monitor high- and low-risk patients. Among the high-risk patients, 19.1% received monitoring within 1 year of exceeding the 500 g cumulative dose threshold. Additionally, 14.4% (64 of 445) of the high-risk patients received annual monitoring examinations after surpassing the threshold. Furthermore, 29.2% and 31.0% of the high- and low-risk patients, respectively, underwent monitoring within a 1-year timeframe before the end of the study, suggesting ongoing vigilance in monitoring in less than one-third of patients in both groups.

**Table 3:** Descriptive statistics of screening practices performed for monitoring examinations in high- and low-risk patients.

Characteristics	High-risk (n = 445)	Low-risk (n = 22,185)
Frequencies of monitoring		
No. of high-risk users receiving any monitoring examinations after 2018/No. of high-risk users	234 (52.6%)	10,963 (49.4%)
No. of patients receiving monitoring within 1 year from exceeding 500 g cumulative dose*/no. of high-risk users	85 (19.1%)	N/A
No. of patients receiving annual monitoring from exceeding 500 g cumulative dose*/no. of high-risk users	64 (14.4%)	N/A
No. of patients receiving recent monitoring during 1-year frame before the study end date	130 (29.2%)	6870 (31.0%)
Modalities used		
Funduscopy/fundus photography	232 (98.7%)	10,874 (99.2%)
Optical coherence tomography	133 (56.6%)	5774 (52.7%)
Automated visual fields	60 (25.5%)	2160 (19.7%)
Fundus autofluorescence	20 (8.5%)	770 (7.0%)
Multifocal electroretinogram	2 (0.9%)	80 (0.7%)
Others^†^	33 (14.0%)	1630 (4.4%)

*N/A* not applicable.

*Monitoring examinations performed within 1 year from when the cumulative dose exceeded 500 g.

^†^Others include fluorescein angiography, OCT angiography, and electroretinography.

The table also provides data on the modalities employed for monitoring examinations. Funduscopy/fundus photography was the most frequently used modality, employed in 98.7% of the cases, followed by OCT in 56.6% of the cases. Automated visual fields and FAF were used in 25.5% and 8.5% of the cases, respectively, whereas multifocal electroretinography constituted a smaller fraction of the monitoring practices at 0.9%.

**Figure 3:**
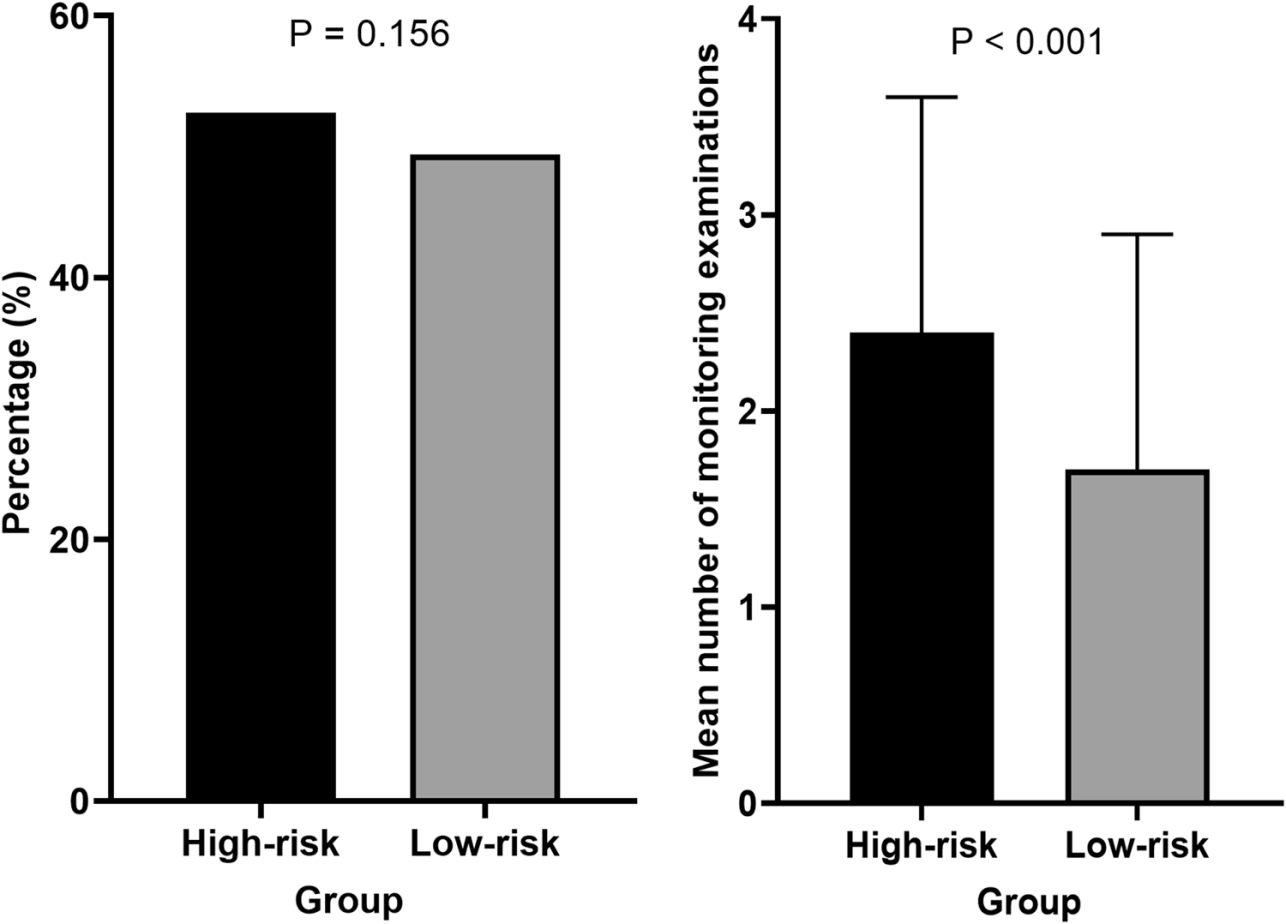
Comparison of monitoring examination rates and total examination counts between high-risk and low-risk patients from 2018 to 2021. Error bars indicate standard deviation.

Figure 3 compares the frequency of ophthalmic examinations between high- and low-risk patient groups from 2018 to 2021. Overall, 234 of the 445 high-risk patients (52.6%) underwent monitoring examinations during the study period and 10,963 of the 22,185 (49.4%) low-risk patients were examined. The difference between the two groups were not statistically significant (P = 0.156). This figure also illustrates the number of monitoring examinations conducted from 2018 to 2021, highlighting a significant difference between high- and low-risk users (2.4 vs. 1.7; P < 0.001).

### Trends in screening practices and comparison between low- and high-risk patients

Table 4 provides a comparison of the annual percentages of high- and low-risk patients undergoing monitoring examinations from 2018 to 2021. In 2018, the data revealed that 22.7% of high-risk patients underwent these examinations, while similar percentage, 27.1% of low-risk patients underwent the same screening, showing no significant difference between the groups (P = 0.454). The percentages of high- and low-risk patients who underwent retinal examinations increased gradually, although this was only statistically significant in low-risk patients (P < 0.001 by the Cochran-Armitage Trend Test). However, no significant differences were observed in the annual frequencies between the groups.

**Table 4:** Comparison of annual percentage of patients receiving ophthalmic (retinal) examinations between high- and low-risk groups and among years.

Year	High-risk patients	Low-risk patients	*P*
2018	22/97 (22.7%)	3746/13,833 (27.1%)	0.454
2019	45/160 (28.1%)	5107/17,416 (29.3%)	0.806
2020	71/284 (25.0%)	5804/20,530 (28.3%)	0.357
2021	130/445 (29.2%)	6870/22,185 (31.0%)	0.563
P value among years^†^	0.376	< 0.001	

^†^Cochran-Armitage trend test.

The annual percentage of patients receiving appropriate imaging for PPM was compared between the high- and low-risk groups from 2018 to 2021 (Supplemental Table 2). The data revealed a notable shift in monitoring practices over the study period, with high- and low-risk patients gradually undergoing these screenings at higher rates. The P-values among years, calculated using the Cochran-Armitage Trend test, demonstrated a statistically significant increase in examination rates over time for both high-risk (P = 0.004) and low-risk (P < 0.001) patients. However, there were no significant differences in the annual frequencies between the high- and low-risk patients from 2018 to 2021 (all P > 0.05).

### Association between drug exposure groups and cumulative incidences of maculopathy

Table 5 presents the results of the multivariate Cox regression analyses conducted to evaluate the association between various factors, including age, sex, the presence of DM and HTN, and drug exposure, and the development of macular conditions. The analysis revealed significant associations between age and both outcomes, with older age being associated with an increased risk. Female sex was also associated with a higher risk of maculopathy excluding common macular diseases, whereas the presence of DM and HTN was not significantly associated with either outcome. The relationship observed between drug exposure and the development of both overall macular degenerations and maculopathy excluding common macular diseases was particularly significant. Both low- and high-risk groups exhibited elevated hazard ratios (HRs) for overall macular degeneration (1.27 [95% confidence interval (CI) 1.03–1.58] and 1.38 [95% CI 1.12–1.71], respectively) and maculopathy excluding common macular diseases (1.55 [95% CI 1.13–2.12] and 1.66 [95% CI 1.22–2.27], respectively), compared to the minimal exposure group.

**Table 5:** Multivariate Cox regression analysis for overall macular degeneration and maculopathy excluding common macular diseases in pentosan polysulfate users.

Factor	Overall macular degeneration		Maculopathy excluding common macular diseases	
	HR (95% CI)	P	HR (95% CI)	P
Age	1.06 (1.06–1.06)	< 0.001	1.05 (1.05–1.06)	< 0.001
Sex		0.289		0.008
Male	1 (ref)		1 (ref)	
Female	1.02 (0.98–1.06)		1.07 (1.02–1.13)	
DM	1.06 (0.77–1.44)	0.736	1.17 (0.77–1.76)	0.470
HTN	0.85 (0.63–1.14)	0.273	0.99 (0.66–1.46)	0.942
Drug exposure group		< 0.001		0.001
Minimal exposure	1 (ref)		1 (ref)	
Low-risk	1.27 (1.03–1.58)		1.55 (1.13–2.12)	
High-risk	1.38 (1.12–1.71)		1.66 (1.22–2.27)	

## Discussion

In recent years, the emergence of progressive maculopathy, characterized by retinal pigmentation and outer retinal atrophy, in patients undergoing PPS has sparked considerable concern because of its potential to cause permanent vision impairment. The development of PPM has been linked to drug exposure, necessitating careful attention to maculopathy screening, particularly for high-risk patients exposed to elevated drug levels^3,4,6,7,9,10,19^. We aimed to assess the risk and screening practices for maculopathy in patients using PPS and evaluate their association with different risk groups categorized by PPS exposure levels. Using the data from a large number of PPS users, this study evaluated whether high-risk patients underwent monitoring examinations more frequently and appropriately than low-risk patients.

The analysis of the number of new and cumulative high-risk patients (Table 2) from 2018 to 2021 revealed a consistent and significant increase. This trend mirrors our previous findings, indicating a continuous increase in the annual utilization of PPS^13^. Particularly, the exponential increase in the number of patients at high risk of PPM should be noted. While the absence of reported cases of PPM in Korea may be attributed to the relatively small number of high-risk users in the country thus far, given that the drug was approved for use relatively recently (2003), the evident surge in the number of high-risk users suggests that this issue could emerge as a significant concern for the Korean population in the near future.

The observed substantial disparity in the incidence of maculopathy among the three exposure groups, as illustrated in Fig. 2, underscores the association between cumulative PPS dosage and the development of maculopathy. The higher cumulative incidence of overall macular degeneration and maculopathy excluding common macular diseases in high-risk patients compared with their low-risk counterparts suggests a cumulative dose-dependent risk of maculopathy. This also justifies our method to categorize patients into high and low-risk groups based on 500 g cumulative dose of PPS, the criterion used for separating PPS users into risk groups in a previous study^6^. However, Ludwig et al. observed no association between PPS exposure and subsequent diagnosis of toxic maculopathy, which appears to contradict our findings^4^. Nonetheless, in that study, a significant association was observed between any maculopathy and PPS exposure levels, which is consistent with our findings. This could be attributed to PPM being diagnosed with other causes of maculopathy in real-world practices, although further research is needed to clarify this aspect. Similar dose–response relationships between drug exposure and the incidence of PPM were reported in multiple studies^20,21^; accordingly, these findings underscore the importance of risk stratification based on cumulative dose and emphasize the need for heightened awareness and recognition of toxicity by screening physicians for high-risk patients.

Furthermore, the results of our multivariate Cox regression analyses revealed notable associations between various factors and the development of maculopathy among PPS users. Specifically, older age was significantly associated with an increased risk of overall macular degeneration and maculopathy excluding common macular diseases. Importantly, our analysis revealed a significant relationship between drug exposure groups and the development of both overall macular degeneration and maculopathy excluding common macular diseases after controlling for several confounders. These findings highlight the importance of considering age and drug exposure levels when assessing the risk of maculopathy among PPS users.

However, our results showed that only approximately half of the PPS users, regardless of high- or low-risk for PPM, received monitoring examinations during the 4-year period between 2018 and 2021. This indicated that the other half of the patients (47.4% of high-risk users) remained unscreened for PPM. This is particularly problematic in high-risk users because the condition may lead to permanent visual loss and outer retinal damage if left undetected. Furthermore, the average annual frequency in which monitoring examinations are conducted was relatively low, with each patient receiving an average of 2.4 examinations (0.6 per year) between 2018 and 2021. Although this was significantly greater than that in low-risk patients (1.7), our findings emphasize that even among those who underwent monitoring, the frequency was not sufficient to detect early PPM given the cumulative dose-related development and progressive nature of the condition. The identification of 19.1% of high-risk patients who received their initial monitoring examinations within one year of exceeding the 500 g cumulative dose threshold suggests that there was a delay in initiating these screenings in a substantial portion of high-risk users, which could miss early intervention opportunities. Moreover, Table 3 demonstrates a notable standard deviation (1.2) in the mean number of monitoring examinations for high-risk patients, indicating substantial data variability. The lack of firmly established organizational guidelines may contribute to the wide variation observed in the frequency of monitoring examinations among high-risk patients, underscoring the imperative need for the establishment of screening guidelines.

Accordingly, promoting early screening immediately after exceeding the 500 g cumulative dose threshold and enhancing the frequency of monitoring examinations by regular monitoring are of utmost importance for high-risk users. Given the exponential increase in high-risk users over the years, this study highlights the pressing need for more proactive measures and awareness campaigns to ensure that individuals at high risk for PPM receive timely and regular screenings. Collaboration between prescribers and eye care specialists is pivotal for raising awareness and ensuring that monitoring practices are performed at least annually in high-risk users, thereby reducing the risk of vision impairment and vision-threatening complications. These findings also necessitate the formulation of standardized guidelines encompassing risk assessments based on drug exposure levels, frequency and timing of screening, and modalities used for screening, similar to the established guidelines for hydroxychloroquine retinopathy outlined by the American Academy of Ophthalmology^14^.

The findings regarding the modalities used for monitoring examinations (Table 3) revealed valuable insights into the screening practices employed. Funduscopy/fundus photography was the dominant modality utilized in 98.7% of cases, which had limited sensitivity. OCT was used in 56.6% of the cases, underscoring its increasing significance in the sensitive detection of PPM, although its adoption could be higher. Remarkably, the utilization of FAF was infrequent, as it was used only in 8.5% of high-risk users, resulting in a low percentage of high-risk users receiving appropriate imaging for PPM (Supplemental Table 2): 7.2% in 2018 to 18.0% in 2021. The ability of OCT to provide detailed structural information on a cross-sectional image of the macula and the role of FAF in the detection of RPE changes make them valuable tools for identifying subtle macular changes in eyes with PPM^5,10^. In light of early detection of maculopathy, our findings suggest a growing recognition and application for these sensitive modalities in monitoring high-risk PPS users among screening physicians; nevertheless, the percentage of those receiving appropriate screening (< 20%) indicates a need for further enhancement.

The trends in screening practices and comparisons between low- and high-risk patients (Fig. 2 and Table 4) provide a comprehensive overview of the monitoring examination frequencies over the study period. The data revealed a significant increase in the percentage of high-risk patients who underwent retinal examinations over the study period, reaching 29.2% by 2021. The overall shift in monitoring practices implies an increased awareness and vigilance regarding PPM screening. However, there were no significant differences in the annual frequencies between the different risk groups (Table 4), suggesting that more proactive and focused monitoring practices for high-risk users are more beneficial for mitigating the risk of PPS-related maculopathy.

Although our study provides valuable insights into screening practices and their association with PPS exposure in high-risk patients, there are some limitations to consider when interpreting the data. The retrospective nature of the study design and identification of unknown screening practices using operational definitions from the comprehensive medical claims database might have introduced selection bias, as the definitions for screening practices and high-risk patients may be incomplete and not well established for PPM^5,8,10,11^. Furthermore, our classification resulted in notable variability in the distribution of patients across the three exposure groups. Specifically, the number of high-risk patients who warranted special attention in clinical practice and risk assessment was relatively limited. This finding underscores the importance of future studies involving larger numbers of high-risk users to ensure robust and reliable conclusions. Additionally, we could not exclude ophthalmic examinations performed on participants for purposes other than toxicity screening. However, regardless of the purpose of ophthalmic examinations, performing appropriate tests is crucial for detecting PPM, as these tests may sensitively capture its characteristic features. Furthermore, this study focused only on the South Korean population, which may limit the generalizability of the findings to other regions with different healthcare systems and patient demographics.

Moreover, additional confounding factors and underlying causes could have contributed to maculopathy in our study. We conducted multivariate analyses to account for potential confounders and aimed to minimize other causes by including diagnostic codes for maculopathy that occurred only after PPS initiation. Despite these efforts, complete exclusion of confounding factors or other causes of maculopathy might not have been achieved. Finally, our study employed broader categories to encompass PPM within our database because patients with PPM might have been diagnosed with various forms of macular degeneration. This could be due to the fact that the diagnostic code for 'toxic maculopathy' was available for a relatively limited duration during the study period. Additionally, PPM might have been recognized as other types of macular degenerations by screening physicians in Korea, where PPM cases have not been reported and awareness about the entity is limited. While our approach, involving aggregates of maculopathies of interest, aligns with the previous study by Ludwig et al.^4^, it is essential to interpret our findings with caution.

In conclusion, our study suggests the need for standardized and targeted screening practices for high-risk patients and highlights the ophthalmologists’ role in performance of appropriate tests. To reduce the number of unscreened high-risk PPS users, enhancements in referral systems to ophthalmologists and increased awareness of associated risks are necessary, particularly for high-risk users. Establishing guidelines or a structured approach to screening is imperative to ensure timely detection and intervention, thereby averting irreversible vision impairment associated with PPM.

### Supplementary Information

Supplementary Information.

## Data Availability

The datasets used and/or analysed during the current study available from the corresponding author on reasonable request.

## References

[1] Patnaik SS (2017). Etiology, pathophysiology and biomarkers of interstitial cystitis/painful bladder syndrome. Arch. Gynecol. Obstet..

[2] Nickel JC (2005). Randomized, double-blind, dose-ranging study of pentosan polysulfate sodium for interstitial cystitis. Urology.

[3] Pearce WA, Chen R, Jain N (2018). Pigmentary maculopathy associated with chronic exposure to pentosan polysulfate sodium. Ophthalmology.

[4] Ludwig CA, Vail D, Callaway NF, Pasricha MV, Moshfeghi DM (2020). Pentosan polysulfate sodium exposure and drug-induced maculopathy in commercially insured patients in the United States. Ophthalmology.

[5] Lindeke-Myers A, Hanif AM, Jain N (2022). Pentosan polysulfate maculopathy. Surv. Ophthalmol..

[6] Vora RA, Patel AP, Melles R (2020). Prevalence of maculopathy associated with long-term pentosan polysulfate therapy. Ophthalmology.

[7] Wang D (2020). Pentosan-associated maculopathy: Prevalence, screening guidelines, and spectrum of findings based on prospective multimodal analysis. Can. J. Ophthalmol..

[8] Wang D (2021). Pentosan polysulfate maculopathy: Prevalence, spectrum of disease, and choroidal imaging analysis based on prospective screening. Am. J. Ophthalmol..

[9] Hanif AM (2019). Phenotypic spectrum of pentosan polysulfate sodium-associated maculopathy: A multicenter study. JAMA Ophthalmol..

[10] Abou-Jaoude M, Fraser C, Maldonado RS (2021). Update on maculopathy secondary to pentosan polysulfate toxicity. Curr. Opin. Ophthalmol..

[11] Abou-Jaoude MM (2021). New insights into pentosan polysulfate maculopathy. Ophthalm. Surg. Lasers Imaging Retina.

[12] Higgins K (2020). Identification of patients with pentosan polysulfate sodium-associated maculopathy through screening of the electronic medical record at an academic center. J. Ophthalmol..

[13] Kim J, Kwon HY, Kim JH, Ahn SJ (2023). Nationwide usage of pentosan polysulfate and practice patterns of pentosan polysulfate maculopathy screening in South Korea. Ophthalmol. Retina.

[14] Marmor MF (2016). Recommendations on screening for chloroquine and hydroxychloroquine retinopathy (2016 revision). Ophthalmology.

[15] Yusuf IH, Foot B, Lotery AJ (2021). The royal college of ophthalmologists recommendations on monitoring for hydroxychloroquine and chloroquine users in the United Kingdom (2020 revision): Executive summary. Eye.

[16] Kim J, Kim KE, Kim JH, Ahn SJ (2023). Practice patterns of screening for hydroxychloroquine retinopathy in South Korea. JAMA Netw. Open.

[17] Barnett JM, Jain N (2022). Potential new-onset clinically detectable pentosan polysulfate maculopathy years after drug cessation. Retin. Cases Brief Rep..

[18] Kalbag NS, Maganti N, Lyon AT, Mirza RG (2021). Maculopathy secondary to pentosan polysulfate use: A single-center experience. Clin. Ophthalmol..

[19] van Ophoven A, Vonde K, Koch W, Auerbach G, Maag KP (2019). Efficacy of pentosan polysulfate for the treatment of interstitial cystitis/bladder pain syndrome: Results of a systematic review of randomized controlled trials. Curr. Med. Res. Opin..

[20] Philip AM, Wannamaker KW, Miller DM (2023). Prevalence and dose dependency analysis of pentosan polysulfate sodium maculopathy. Ophthalmic Epidemiol..

[21] Bae SS, Sodhi M, Maberley D, Kezouh A, Etminan M (2022). Risk of maculopathy with pentosan polysulfate sodium use. Br. J. Clin. Pharmacol..

