# Supplementary material for: Screening practices and risk assessment for maculopathy in pentosan polysulfate users across different exposure levels
Source: Sci Rep. 2024 May 17;14:11270. doi: 10.1038/s41598-024-62041-y (PMC11101426; doi:10.1038/s41598-024-62041-y)
Supplement: Supplementary file 1 — Supplementary Information. [file 41598_2024_62041_MOESM1_ESM.docx]

**Supplementary Online Content**

**Supplemental Table 1.** Definitions and Korean Classification of Disease (KCD) codes for categories of overall macular degeneration and maculopathy excluding common macular diseases  **Supplemental Table 2.** Comparison of annual percentage of patients receiving appropriate imaging (optical coherence tomography or fundus autofluorescence) for pentosan polysulfate maculopathy between low-risk and high-risk groups and among years

This supplementary material has been provided by the authors to give readers additional information about their work.

**Supplemental Tables**

**Supplemental Table 1.** Definitions and Korean Classification of Disease (KCD) codes for categories of overall macular degeneration and maculopathy excluding common macular diseases

| **Category** | **KCD codes** | **Specific conditions** |
| --- | --- | --- |
| Overall macular degeneration | **H31.0**  **H35.3**  (including H35.30,  H35.31,  H35.32,  H35.33,  H35.34, | Macular scars of the posterior pole  Degeneration of macula and posterior pole  Nonexudative age-related macular degeneration  Exudative age-related macular degeneration  Angioid streaks of macula  Macular cyst, hole, or pseudohole  Cystoid macular degeneration |
|  | H35.35,  H35.36,  H35.37,  and H35.39) | Drusen of macula  Puckering of macula  Toxic maculopathy (available up to Dec. 31, 2020)  Unspecified macular degeneration |
| Maculopathy excluding common macular diseases* | **H35.37** | Toxic maculopathy (available up to Dec. 31, 2020) |
|  | **H35.39** | Unspecified macular degeneration |

*Common macular diseases include drusen, age-related macular degeneration, epiretinal membrane (puckering of macula), macular hole, and other specific degenerations.

**Supplemental Table 2.** Comparison of annual percentage of patients receiving appropriate imaging (optical coherence tomography or fundus autofluorescence) for pentosan polysulfate maculopathy between low-risk and high-risk groups and among years

| **Year** | **High-risk patients** | **Low-risk patients** | **P** |
| --- | --- | --- | --- |
| 2018 | 7/97 (7.2%) | 1,487/13,833 (10.7%) | 0.307  0.599  0.245  0.513 |
| 2019 | 18/160 (11.3%) | 2,234/17,416 (12.8%) |  |
| 2020 | 31/284 (10.9%) | 2,795/20,530 (13.6%) |  |
| 2021 | 80/445 (18.0%) | 3,681/22,185 (16.6%) |  |
| P value among years^†^ | 0.004 | <0.001 |  |

^†^Cochran-Armitage Trend Test
